# How Effective Are Telephone and Video Consultations in Shoulder and Elbow Clinics? Analysis Using an Objective Scoring Tool

**DOI:** 10.7759/cureus.17380

**Published:** 2021-08-23

**Authors:** Vusumuzi Sibanda, Ifeanyi Onubogu, Marjan Raad, Siddharth Virani, Jai Relwani

**Affiliations:** 1 Trauma and Orthopaedics, William Harvey Hospital, Ashford, GBR; 2 Trauma & Orthopaedics, Darent Valley Hospital, Dartford, GBR

**Keywords:** virtual consultations, telemedicine, covid-19, video consultations, ashford clinic letters score, outpatient clinic

## Abstract

Introduction: The coronavirus disease 2019 (COVID-19) pandemic resulted in disruptions of clinical services, which saw more clinics being conducted as telephone and videos. The study aimed to assess and compare the effectiveness of consultations, that is, telephone, video, and face-to-face (F2F) in a shoulder and elbow clinic.

Methods: A total of 84 clinic letters from a shoulder and elbow clinic at a district general hospital were analysed using the Ashford Clinic Letter Scoring System. Of these, 30 were F2F, 30 were telephone, and 24 were video consultations. The letters were analysed and scored based on four parameters, that is, whether a working diagnosis was formulated, relevant investigations were requested or available, a clear management plan was formulated, and whether the consultation was deemed valuable by both the clinician and patient.

Results: The mean score (out of a total possible of eight) for F2F was 7.967, 7.667 for video, and 7.333 for telephone consultations. Amongst new referrals, F2F performed the best, followed by video with telephone consultations scoring the lowest. With follow-up referrals, the performance of telephone and video consultations was similar but more inferior compared to F2F. Videos performed nearly as well as F2F when it came to formulating treatment plans for patients.

Conclusion: F2F consultations remain the gold standard in a shoulder and elbow clinic; however, careful stratification of patients into video, telephone, and F2F clinics can help in optimal delivery of care. These findings can be applied to other surgical specialties and medicine in general. Virtual clinics are viable and potentially cost-effective options to the traditional F2F.

## Introduction

Telemedicine has rapidly evolved and has been adapted by different healthcare systems across the world and its use has increased during the coronavirus disease 2019 (COVID-19) pandemic [1]. During the first wave of COVID-19 in the United Kingdom in March 2020, The British Orthopaedic Association gave guidance that, if possible, outpatient appointments should be virtual [2,3]. Even after the end of the first wave of COVID-19 in the UK, telephone and video consultations remained popular in the trauma and orthopaedic department. Their use continued into the second wave of the COVID-19 infection in October-November 2020.

Although many studies demonstrated the effectiveness and usefulness of telemedicine, the data are sparse on the objective efficacy of telemedicine compared to face-to-face consultations.

This study sought to assess how effective virtual consultations are in comparison to traditional face-to-face consultations using an objective metric. We used the Ashford Clinic Letter Scoring System (ACLS) to score clinic letters in an elective shoulder and elbow orthopaedic clinic. The ACLS tool has proven to be reliable, reproducible, and concise, which aids in objectively assessing and auditing the quality and efficacy of consultations [3,4].

## Materials and methods

Consultants run elective shoulder and elbow outpatient clinics with the assistance of middle-grade registrars and clinical fellows, as well as dedicated upper limb physiotherapists. After each consultation, the clinician is required to document the consultation, and a copy of the letter is sent to the patient’s general practitioner (GP) and the patient. In the study, we retrospectively analysed 84 clinic letters, 30 of which were face-to-face (F2F), 30 telephone, and 24 video consultations. The letters were taken from patients seen from March to April 2021. 

The video consultations were conducted using accuRx (accuRx Ltd, London, England) and Attend Anywhere (Attend Anywhere, Melbourne, Australia) software, while telephone consultations were done using the National Health Service (NHS) trust telephones. The efficacy of the consultations was assessed using the Ashford Clinic Letter Scoring (ACLS) system. The ACLS is a valid, reliable tool used to assess the effectiveness of a clinical consultation based on four parameters which are:

1. whether a precise working diagnosis was made;

2. all relevant investigations were requested or available;

3. a clear management plan was formulated; and

4. whether the consultation was deemed valuable by both the clinician and the patient.

Each parameter is given a maximum of two points giving a maximum possible score of eight (Table 1). Two independent assessors scored the letters on the basis of guidelines of the ACLS. A mean of the two scores of the two assessors was taken as the finals score (Table 2).

**Table 1:** The Ashford Clinic Letters Score *When a registrar/fellow reviews the patient [3].

The Ashford Clinic Letters Score	
Parameter	Score
A - Diagnosis	
Clear working diagnosis made	2
Face-to-face review would improve the diagnosis	1
No diagnosis	0
B - Investigations	
All relevant investigations are done and available	2
Tests/imaging done but not available at time of consultation	1
Clear which investigations are needed and these have been ordered	1
Not clear what is needed at this stage	0
C - Formulation of the treatment plan	
Treatment plan made or formulated	2
Face-to-face review needed to clarify treatment plan	1
Consultant input needed within three weeks for a treatment plan*	1
No treatment plan could be agreed	0
D - Consultation was valuable communication	
Yes	2
No, but the doctor-patient relationship is intact	1
No, and future treatment will be difficult due to impaired relationship	0
Total	….

**Table 2:** Guidelines on allotting scores to the Ashford Clinic Letters Score *Although the score here is 2, the score in component B would be 1 [3].

Guidelines on allotting scores to the Ashford Clinic Letters Score	
Parameter	Score
A - Making a diagnosis	
Clear working diagnosis made based on history and investigations available	2
Patient improving postoperatively	1
The examination would help confirm the region of the pathology/inconclusive examination	1
Post-operative patient worsening or not improving	1
Cannot identify the region of pathology	0
B - Investigations	
All needed investigations are done and available	2
Follow-up radiographs/blood are needed in a few months	2
Relevant investigations (MRI, CT, Bone Scans, etc.) were requested for	1
Radiographs/blood needed within three weeks	1
Unsure what is needed	0
C - Treatment plan	
Listed for surgery	2
Discharged	2
Routine post-op follow-up arranged	2
Follow-up to review pertinent investigations	2
Follow-up to see how the patient is “getting along”	1
A treatment plan cannot be agreed	0
D - Value of the consultation	
Patient and doctor satisfied by the outcome	2
The patient would have preferred face-to-face consultation	1
Surgeon/patient unhappy/dissatisfied	1
Consultation made future treatment more difficult due to relationship breakdown	0

The scores were then entered into an Excel spreadsheet (Microsoft Corporation, Redmond, Washington.) The patients were divided into new and follow-up patients under each category (F2F, telephone, and video). The mean score for each category was calculated along with the standard deviations. The ANOVA test was applied to assess any statistically significant difference between the three groups assuming 95% confidence intervals.

## Results

**Figure 1:**
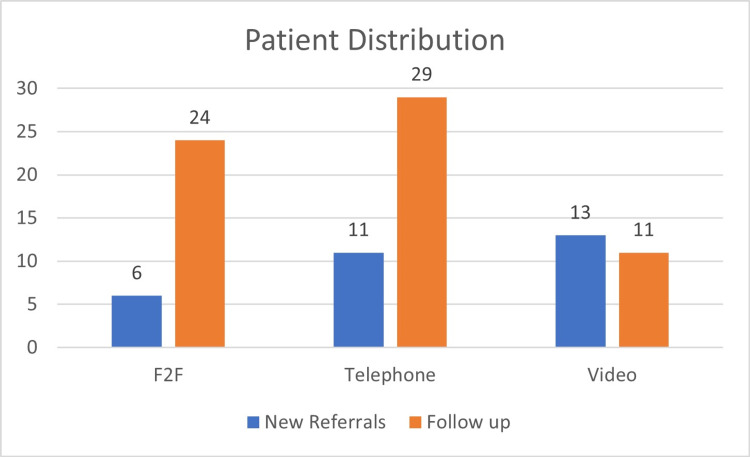
Patient distribution

Of the 30 face-to-face consultations, six were new referrals, and 24 were follow-ups (16 routine follow-ups and eight post-op follow-ups). Of the telephone consultations, 11 were new referrals and 19 were follow-ups (13 routine follow-ups and six post-op follow-ups). Regarding the 24 video consultations, 13 were new referrals and 11 were follow-ups (nine routine follow-ups and two post-op follow-ups) (Figure 1).

**Figure 2:**
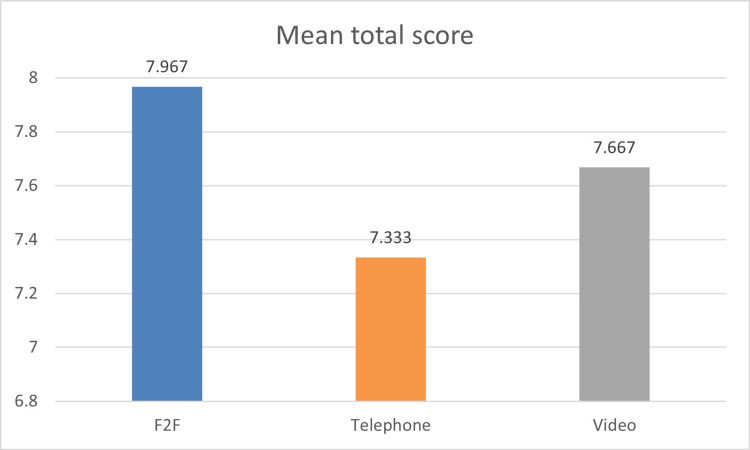
Mean total score

The average total score (out of a possible score of eight) for F2F was 7.967, while it was 7.667 for video and 7.333 for telephone consultations (Figure 2). On performing the ANOVA test assuming 95% confidence intervals between the three groups, the differences were found not to be statistically significant (p < 0.05) (p = 0.0091). Further, on performing a pairwise assessment, a significant difference (p < 0.05) was found between the telephone and face-to-face consultations, but this was not noticed amongst the other pairs of videos versus face-to-face consultations and telephone versus video (p = 0.33 and p = 0.25, respectively).

**Figure 3:**
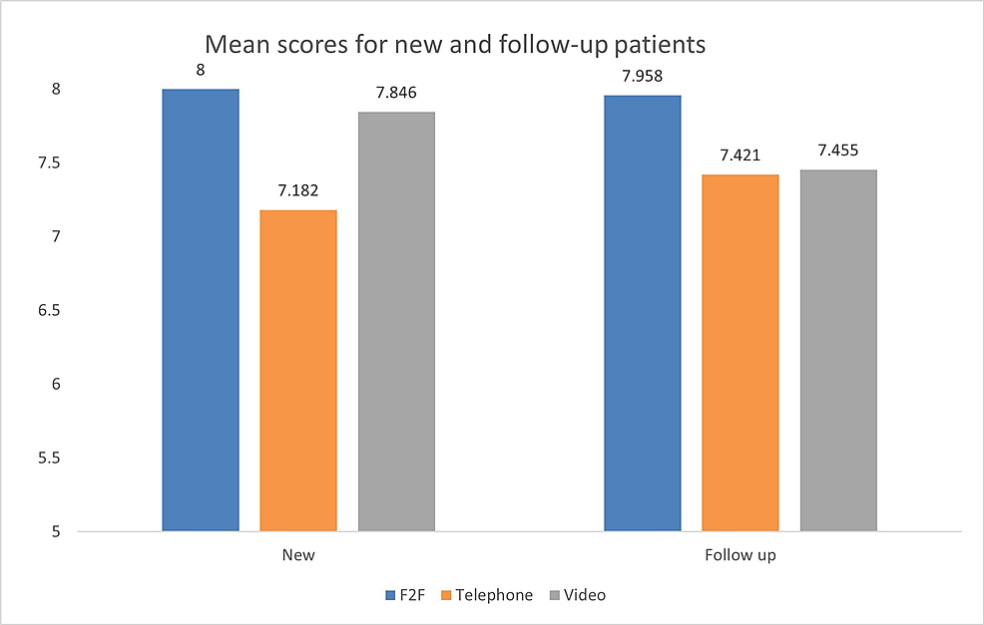
Mean scores for new and follow-up patients

Among the new referrals, face-to-face consultations performed the best, followed by video consultations, with the telephone consultations scoring the lowest. With follow-up referrals, the performance of telephone and video consultations was similar but more inferior compared to face-to-face consultations, although this was not statistically significant (Figure 3).

**Figure 4:**
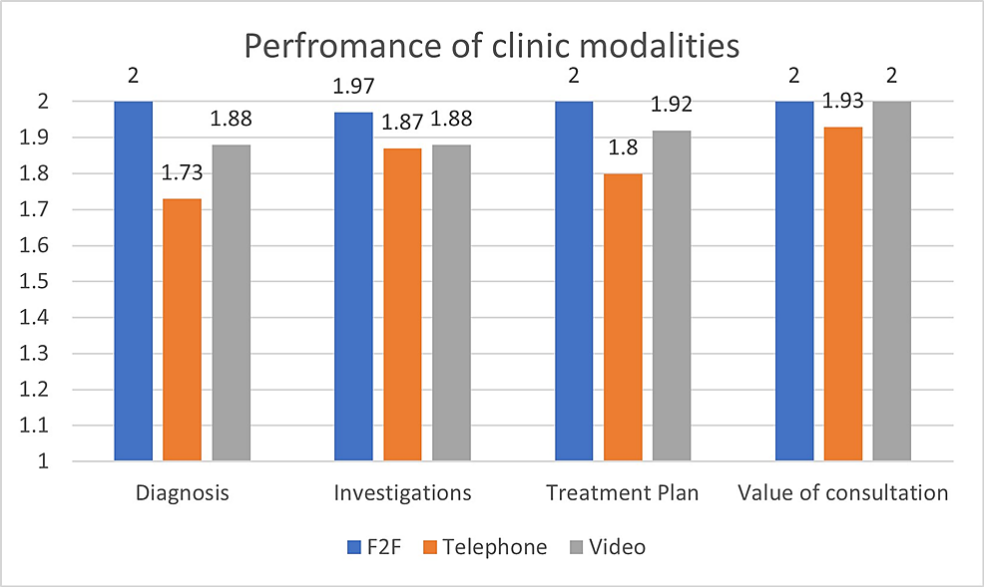
Performance of clinic modalities across each parameter

On analysis, video consultations performed nearly as well as face-to-face consultations when it came to formulating a treatment plan for patients (score 1.917). However, all three modes added value in the progress of the patients’ journey in specialist care (Figure 4).

## Discussion

As the COVID-19 pandemic gripped the world, healthcare systems across the globe started to struggle with the burden of patients requiring hospital admissions [5]. The COVID-19 pandemic has served as a catalyst in advancing telehealth technology and increasing its utilisation and integration into the current clinical environment [6]. Adaptation is a necessity during this crisis, and telemedicine has taken centre stage across various medical specialities and appears to provide solutions to some of the problems faced. Many trauma and orthopaedic teams have adopted remote consultations with telephone clinics, virtual fracture clinics, and video consultations [7,8]. Purported benefits of telemedicine include decreased travel burden, patient convenience, cost-savings [9,10].

Grandizio et al. carried out a prospective study of telemedicine program implementation after upper extremity surgery. They found that their program decreases travel burdens associated with conventional in-clinic appointments, significantly decreases visit times without decreasing patient satisfaction, and the ability to recognise early postsurgical complications was not compromised by utilising this technology, even during their early experience [11]. Siow et al. carried out several strategies to ensure they were tailored around the telehealth visit. These included: postponing long-term follow-up visits, having sutures or staples removed by a home health or skilled nursing facility registered nurse, having patients obtain pertinent imaging before the visit, and ensuring that patients have access to mobile devices and internet connectivity [12].

Regarding patient satisfaction, Sharareh and Schwarzkopf found that patients receiving telemedicine visits after total joint arthroplasty reported higher satisfaction with fewer calls to the clinic staff [13]. Sathiyakumar et al. compared patient satisfaction between telemedicine and in-person follow-up appointments for orthopaedic trauma patients. A total of 24 patients were enrolled and randomised to each group with a total of four follow-up appointments during a six-month period. The authors reported no difference in patient satisfaction between telemedicine and in-person clinic visits; however, no one in the telemedicine group took time off from work and spent markedly less time on their visits compared with the clinic follow-up group, with most patients (75%) agreeing to future follow-up visits with telemedicine [14]. Rizzi et al. published a study documenting patients and orthopaedic surgeons having high levels of satisfaction with telehealth encounters during the COVID-19 pandemic [15].

Our study shows that video consultations are equally effective as face-to-face consultations according to the Ashford Clinic Letter Scoring system. This applies to both new referrals as well as follow-up. Telephone consultations proved to be a reliable option for consultation for follow-up patients.

Our study is the first paper to quantify the efficacy of different modes of clinical consultations in shoulder and elbow surgery. This study uses a clinic letter as a surrogate to the actual consultation and hence lack of detailed documentation could affect the accuracy of the score. However, this is partially mitigated by the same team conducting consultations for all modes of clinics. The study captures the data from a single team, and including more teams and clinicians could improve the generalisability of the results. Retrospective data collection could be considered a weakness. In addition, there is bias created by stratifying easier/less complex patients in telemedicine and more complex to face to face. Future studies could evaluate the incidence of missed complications such as infections or stiffness as a result of telehealth, analyse the subset of patients who may be more vulnerable to no-shows or technological failures, and conduct patient surveys to determine the factors that contribute to patient preferences for or against the use of telehealth.

## Conclusions

Face-to-face consultations remain the gold standard in a shoulder and elbow clinic; however, technological advances make video consultations a viable alternative. Careful stratification of patients into video, telephone, and F2F clinics can help in the optimal delivery of care. These findings can be applied to other subspecialties of orthopaedics, surgery, and medicine in general, both in developed and developing countries. More resources need to be put into virtual telemedicine clinics as these are viable and potentially cost-effective options to the traditional F2F and can result in a reduction of the number of patients lost to follow-up.
